# Improvement of the Efficiency and Completeness of Neuro-Oncology Patient Referrals to a Tertiary Center Through the Implementation of an Electronic Referral System: Retrospective Cohort Study

**DOI:** 10.2196/15002

**Published:** 2020-03-05

**Authors:** Rocío Fernández-Méndez, Mei Yin Wong, Rebecca J Rastall, Samuel Rebollo-Díaz, Ingela Oberg, Stephen J Price, Alexis J Joannides

**Affiliations:** 1 Department of Clinical Neurosciences University of Cambridge Cambridge United Kingdom; 2 NIHR Brain Injury MedTech Co-operative Department of Clinical Neurosciences University of Cambridge Cambridge United Kingdom; 3 Obex Technologies Cambridge United Kingdom; 4 Neurosurgery Department Cambridge University Hospitals NHS Trust Cambridge United Kingdom

**Keywords:** quality improvement, electronic health records, hospital referral, hospital oncology services

## Abstract

**Background:**

Quality referrals to specialist care are key for prompt, optimal decisions about the management of patients with brain tumors.

**Objective:**

This study aimed to determine the impact of introducing a Web-based, electronic referral (eReferral) system to a specialized neuro-oncology center, using a service-developed proforma, in terms of waiting times and information completeness.

**Methods:**

We carried out a retrospective cohort study based on the review of medical records of referred adult patients, excluding follow-ups. Primary outcome measures were durations of three key phases within the referral pathway and completion rates of six referral fields.

**Results:**

A total of 248 patients were referred to the specialist center during the study period. Median (IQR) diagnostic imaging to referral intervals were 3 (1-5) days with eReferrals, and 9 (4-19), 19 (14-49), and 8 (4-23) days with paper proforma, paper letter, and internal referrals, respectively (*P*<.001). Median (IQR) referral to multidisciplinary team decision intervals were 3 (2-7), 2 (1-3), 8 (2-24), and 3 (2-6) days respectively (*P*=.01). For patients having surgery, median (IQR) diagnostic imaging to surgery intervals were 28 (21-41), 34 (27-51), 104 (69-143), and 32 (15-89) days, respectively (*P*<.001). Proportions of complete fields differed significantly by referral type in all study fields (all with *P*s <.001) except for details of presentation, which were present in all referrals. All study fields were always present in eReferrals, as these are compulsory for referral submission. Depending on the data field, level of completeness in the remaining referral types ranged within 69% (65/94) to 87% (82/94), 15% (3/20) to 65% (13/20), and 22% (8/41) to 63% (26/41) in paper proforma, paper letter, and internal referrals, respectively.

**Conclusions:**

An electronic, Web-based, service-developed specific proforma for neuro-oncology referrals performs significantly better, with shorter waiting times and greater completeness of information than other referral types. A wider application of eReferrals is an important first step to streamlining specialist care pathways and providing excellent care.

**International Registered Report Identifier (IRRID):**

RR2-10.2196/10.2196/15002

## Introduction

### Background

Patients with suspected malignancies of the central nervous system (CNS) should be referred promptly to specialized neurosurgery centers following their initial diagnosis for deciding subsequent management [1]. The limitations of traditional referral methods, including verbal communications and referral letters, can potentially decrease the safety and efficiency of the referral process. Verbal communications can lead to low information retention by the receiver, and hand-written referral letters can be limited by illegibility, unreliable transmission, missing documentation, or incomplete information required to make appropriate decisions [2-4]. Although there are well-established referral pathways for elective and emergency referrals, there are less defined routes for nonelective referrals, especially when these arise from the emergency setting, which is the case for the majority of brain tumor presentations [5]. Moreover, patients with brain tumors and their carers often perceive that their referrals were delayed or they were not seen by the appropriate specialist in the first place [6,7].

Among the most effective proposed strategies to overcome the limitations of traditional referral methods are structured referral sheets or proformas [3,4]. Web-based integrated specialty electronic referrals (eReferrals) are a digital version of a referral system [8], through which referrals are sent securely in real time through the internet, making them instantly available to authorized users. These systems can make the process faster, safer, and easier to follow up, regardless of the health care organization or location of the referrer and receiver. This is particularly relevant in the context of referrals from emergency settings, where there is a high staff turnover and limited time available for nonemergency patient management.

### Objectives

Since April 2016, the neurosurgery unit of 1 of the 16 regional neuroscience centers in England introduced an eReferral system for the referral of its neuro-oncology surgery adult patients for multidisciplinary team (MDT) discussion, as part of a digital quality improvement (QI) project. The aim of this study was to determine the impact of introducing this eReferral system, in terms of reduction of waiting times, that is, improvement of time efficiency, and completeness of the information provided within the referrals.

## Methods

### Neuro-Oncology Referrals and Setting

The study center provides neuro-oncology surgery specialist services for a core population of 3 million plus an extended catchment area of 2.6 million. Patients referred to the study center are adult patients aged 16 years and older, who have a suspected CNS tumor that could benefit from surgery, excluding skull base and spinal tumors (normally managed by a different MDT). Each referral is discussed during weekly MDT meetings, and surgically eligible patients are seen by the neuro-oncology surgery team in the next available clinic. The number of adult patients discussed during MDT meetings ranges within 80 to 120 per month, and approximately 10 to 25 are operated on monthly.

Before 2016, the standard route for nonelective, scheduled referrals to the MDT was fax, email, or post using unstructured letters or structured proformas (jointly referred to as paper referrals). Since April 2016, an eReferral system was introduced to replace paper referrals. The eReferral form was developed with iterative multidisciplinary input, based on its preceding paper proforma and in line with national guidelines [1,9]. The form includes mainly mandatory closed fields and is hosted at the Outcome Registry Intervention and Operation Network (ORION), a secure, Web-based platform, for managing health care data in multiple institutions (Multimedia Appendices 1 and 2; Obex Technologies Ltd, Cambridge). Some fields include a predefined list of possible answers and others are contingent on answers to previous fields. eReferrals can only be submitted when all mandatory fields are completed, and after submission, they are automatically updated in the system and made available to the MDT office in real time. This feature helps to easily determine referral urgency in a timely manner and suitability for the neuro-oncology surgery MDT. Contact details of the referring teams are easily accessible in the eReferrals, making it straightforward to communicate about any outstanding investigations required before the MDT. Internal referrals within the study neuroscience center are mostly done using the electronic hospital records (EHRs) system directly.

Therefore, there were four referral types included in this study: eReferrals via ORION, paper-based referrals using the proforma or a free-text letter, and direct referrals through the EHR. The study period selected included 2 separate months, April and September, to account for seasonal variation of 2 consecutive years, 2016, when the QI project started, and 2017, when the QI project had been in place for over 1 year.

### Study Design and Data Collection

This was a retrospective, cohort study based on the review of different types of medical records. All new adult patients discussed at all weekly MDT meetings during the study period were eligible. New patients included those who were seen for a new condition or a change in a previous pathology. Follow-up cases were excluded, as the discussion for these was usually arranged through internal processes rather than as a new referral.

Patients were identified from the MDT list and their National Health Service (NHS) or hospital numbers were used to link data from different data sources: ORION, EHR, and a national Picture Archiving and Communication System (PACS; Multimedia Appendix 3). ORION data were downloaded automatically from the platform. EHR and PACS data were manually extracted by 2 auditors using a prespecified data collection form. Data quality checks were performed by a different auditor by rechecking data gathered against the original records.

The number and nature of calls or emails sent by referrers to the ORION software team about issues related to the use of the referral portal were obtained from application support logs provided by the software support team.

### Data and Variables Definitions

Data collected included demographics and clinical data including performance status (PS) on referral, initial diagnosis at MDT, and eventual diagnosis following surgery or further investigations (if available); whether key data fields were included in the referral; and key dates within the referral pathway.

PS was based on the Eastern Cooperative Oncology Group/World Health Organization (WHO) PS levels [10]. Diagnoses were grouped depending on the type of tumor, gliomas being grouped into high-grade gliomas (HGGs; WHO grade III-IV) and low-grade gliomas (LGGs; WHO grade I-II). The six fields analyzed for completion rates were agreed by the service MDT as fields that should be included in an ideal referral form or letter based on national guidelines, and included PS, details of presentation, symptom duration, steroid treatment, previous malignancy, and staging computerized tomography (CT) [1]. Key dates extracted included date of earliest diagnostic imaging, defined as the date of the earliest CT or magnetic resonance imaging (MRI) scan available within the 6 months preceding the referral, referral date, and date of MDT decision, defined as the last MDT discussion. Durations of three key phases within the referral pathway were calculated using those dates: earliest diagnostic imaging to referral interval, referral to decision interval, and decision to surgery interval (if applicable).

### Analyses

Data were summarized using relevant descriptive statistics. Continuous variables were compared by referral type, including subanalyses by diagnostic group, using the Kruskal-Wallis equality-of-populations rank test. Pearson chi-squared or Fisher exact test, as appropriate, were used for the comparison of proportions by referral type. Statistical significance was set at the 5% level.

#### Referral Epidemiology

The incidence of average monthly referrals was calculated using population estimates for mid-2016 from the Office for National Statistics [11]. A line chart was used to represent the evolution of the number of referrals per referral type over time. A colored map was created to depict differences in the average incidence of monthly referrals by subregion.

#### Referral Efficiency and Completeness

Durations of the three key phases of interest within the referral pathway and completion rates of each of the six key data fields under investigation were compared by referral type using the relevant statistical test.

#### Missing Data and Outliers

Entry errors were corrected during the data entry quality checks. Missing data were explored for possible trends but not imputed. Outliers were explored in depth, including potential data entry error identification.

This was a nonresearch, digital service QI project, registered as a service improvement program with the Institutional Clinical Audit Department, with project number PRN7723.

## Results

### Sample

There were 248 patients referred during the 4 months of the study period, with monthly numbers of patients discussed at each MDT meeting ranging from 53 to 72. Median (IQR) age was 66.4 (51.3-73.7) years, and the female-to-male ratio was 5:4. Most patients with reported PS had good WHO-PS levels of 0 (94/176, 53.4%) or 1 (48/176, 27.3%; Table 1).

Most common MDT diagnostic groups were meningioma (49/245, 20.0%), metastasis (48/245, 19.6%), and HGG (47/245, 19.2%). In 14.3% (35/245) patients, the diagnosis given at the MDT meeting was *lesion* or *mass*. Eventual diagnosis was available for 32.7% (80/245) patients, of which 95% (75/79) followed a surgical or biopsy procedure. The distribution of these eventual diagnostic groups differed from that of initial MDT diagnostic groups (Table 1), and for some patients, eventual diagnosis differed from their initial MDT diagnosis (Table 2).

Differences between the four referral systems in demographic and clinical data were not statistically significant (Table 1).

Twelve percent (29/248) patients required one or more MDT rediscussions, with no statistically significant differences by referral source (*P*=.53). The main reasons for rediscussion were a request for further investigations locally (25, 86%).

**Table 1:** Characteristics of patients referred to the study neuroscience centers by referral type excluding unknown and unusual referral pathways.

Characteristics			Referral type^a^								*P* value^b^	
			Electronic		Paper proforma		Paper letter		Internal			
Patients, n			83		94		20		41			
Age (years), median (IQR)			70 (56-76)		64 (52-72)		66 (36-76)		58 (47-72)			.15
Female:male ratio			2:2		3:2		4:2		1:3			.72
**WHO^c^ PS^d^ on referral, n (%)^e^**										**.51**		
	PS-0	42 (51)		42 (45)		5 (25)		4 (10)				
	PS-1	24 (29)		18 (19)		0 (0)		4 (10)				
	PS-2	8 (10)		9 (10)		1 (5)		1 (2)				
	PS-3	5 (6)		6 (6)		1 (5)		0 (0)				
	PS-4	4 (5)		0 (0)		0 (0)		0 (0)				
	Not stated	0 (0)		19 (20)		13 (65)		32 (78)				
**Multidisciplinary team meeting diagnosis, n (%)^f^**										**.09**		
	Meningioma	16 (19)		19 (20)		4 (20)		8 (20)				
	HGG^g^ (WHO III-IV)	23 (28)		16 (17)		3 (15)		3 (7)				
	Metastasis	10 (12)		20 (21)		1 (5)		14 (34)				
	Lesion or mass^h^	14 (17)		14 (15)		3 (15)		3 (7)				
	Other CNS^i^ tumor^j^	5 (6)		8 (9)		2 (10)		7 (17)				
	Other nontumor lesion^k^	6 (7)		4 (4)		2 (10)		3 (7)				
	LGG (WHO I-II)^l^	1 (1)		6 (6)		2 (10)		2 (5)				
	Lymphoma	4 (5)		2 (2)		0 (0)		0 (0)				
	Spinal tumor	1 (1)		1 (1)		0 (0)		0 (0)				
	Unknown or unclear	1 (1)		4 (4)		2 (10)		0 (0)				
	Missing/not stated	2 (2)		0 (0)		1 (5)		1 (2)				
Neuro-oncology surgery			34 (41)		37 (39)		9 (45)		13 (32)			.72
**Eventual diagnosis after surgery or further investigations, n (%)**										**.33**		
	HGG (WHO III-IV)	14 (41)		13 (35)		2 (22)		2 (15)				
	Meningioma	2 (6)		3 (8)		2 (22)		4 (31)				
	LGG (WHO I-II)	4 (12)		4 (11)		1 (11)		1 (8)				
	Metastasis	1 (3)		5 (14)		1 (11)		3 (23)				
	Other CNS tumor	2 (6)		2 (5)		0 (0)		0 (0)				
	Lymphoma	0 (0)		1 (3)		0 (0)		1 (8)				
	Non-CNS tumor	0 (0)		0 (0)		0 (0)		1 (8)				
	Unknown or unclear	1 (3)		1 (3)		0 (0)		0 (0)				
	Missing or not stated	10 (29)		8 (22)		3 (33)		1 (8)				

^a^7 referrals initially done as emergency neurosurgery referrals and 3 cases with unknown referral source were excluded.

^b^Excluding cases with unknown or missing values.

^c^WHO: World Health Organization.

^d^PS: performance status.

^e^Eastern Cooperative Oncology Group/WHO performance status levels.

^f^Multidisciplinary team diagnosis: initial/suspected diagnosis following multidisciplinary team meeting discussion.

^g^HGG: high-grade glioma.

^h^Diagnoses included *lesion/s* or *mass*, with or without location mentioned.

^i^CNS: central nervous system.

^j^Diagnoses included *tumor*, *neoplasm*, *glioma*, *ependymoma*, *dermoid*, *craniopharyngioma*, *medulloblastoma*, or *schwannoma*.

^k^Diagnoses included *cyst*, *demyelination*, *bleed*, *infarct*, *abscess*, *inflammatory*, *fungal*, and *herpes*.

^l^LGG: low-grade glioma.

**Table 2:** Distribution (number and percentage) of eventual diagnostic groups after surgery in each initial multidisciplinary team meeting diagnostic group among patients who had surgery.

Initial multidisciplinary team diagnosis	Eventual diagnosis, n (%)								
	High-grade glioma	Low-grade glioma	Lymph	Meningioma	Metastasis	Other central nervous system tumor	Noncentral nervous system tumor	Unknown or unclear	Missing
High-grade glioma	*20 (65)* ^a^	3 (10)	0 (0)	0 (0)	2 (6)	0 (0)	0 (0)	0 (0)	6 (19)
Low-grade glioma	1 (14)	*5 (71)*	0 (0)	0 (0)	0 (0)	0 (0)	0 (0)	0 (0)	1 (14)
Lymph	3 (75)	0 (0)	*1 (25)*	0 (0)	0 (0)	0 (0)	0 (0)	0 (0)	0 (0)
Meningioma	0 (0)	0 (0)	0 (0)	*9 (60)*	0 (0)	1 (7)	0 (0)	0 (0)	5 (33)
Metastasis	0 (0)	1 (10)	1 (10)	0 (0)	*6 (60)*	0 (0)	0 (0)	1 (10)	1 (10)
Other central nervous system tumor	3 (43)	0 (0)	1 (14)	0 (0)	0 (0)	*1 (14)*	0 (0)	0 (0)	2 (29)
Other nontumor lesion	1 (25)	0 (0)	0 (0)	0 (0)	0 (0)	0 (0)	*1 (25)*	0 (0)	2 (50)
Lesion or mass	5 (28)	1 (6)	0 (0)	2 (11)	2 (11)	2 (11)	0 (0)	*1 (6)*	5 (27)
Unknown or unclear	0 (0)	0 (0)	0 (0)	0 (0)	0 (0)	0 (0)	0 (0)	0 (0)	*1 (100)*

^a^Italicized figures represent cases whose final diagnosis was the same as the initial MDT suspected diagnosis.

### Referral Epidemiology

**Figure 1:**
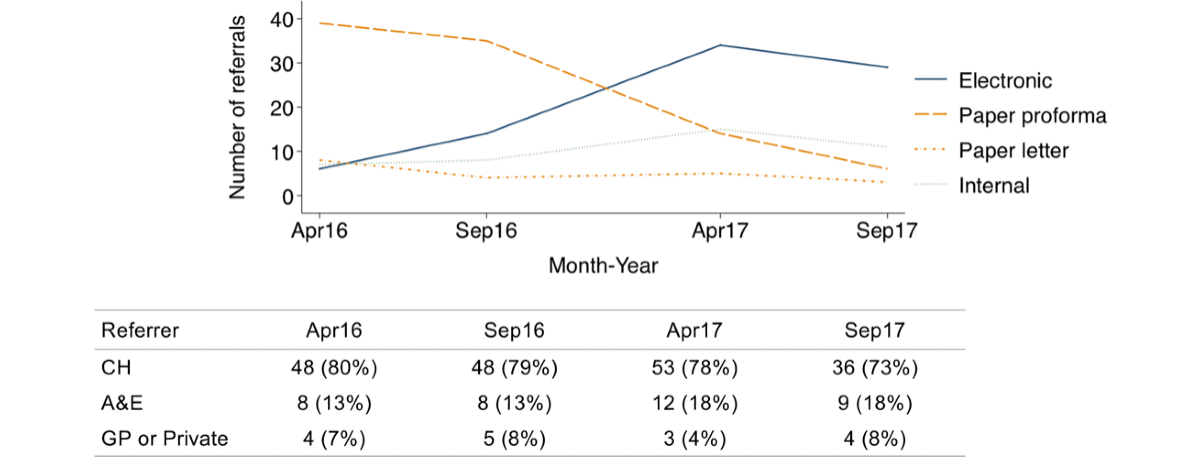
Number of monthly referrals per referral type (top line chart) and distribution of monthly referrals by referrer (bottom table) overtime, in April and September of 2016 and 2017. Patients with nonstandard (initially done as emergency neurosurgery referrals) or unknown referral source are not plotted (n=10). Internal refers to intrahospital referrals within the study center using the hospital’s electronic records system. CH: community hospital; A&amp;E: accidents and emergencies department of the study center; GP: general practice.

During the study period, the use of eReferrals increased since their introduction from 6 (10%) to 29 (55%) referrals per month, whereas monthly paper referrals, with or without proforma, decreased from 47 (77%) to 9 (17%). Internal referrals remained constantly lower than eReferrals, with monthly referrals ranging from 7 (12%) to 15 (21%; Figure 1). In addition, 7 patients had nonstandard referrals (initially referred to as neurosurgical emergencies and usually followed by an appropriate electronic neuro-oncology referral), and 3 patients had an unknown referral source. Most referring centers were district general hospitals (193, 77.8%), followed by referrals within the study neuroscience center (38, 15.3%) and general practice (GP) and private centers (17, 6.9%). These percentages were very similar over time (*P*=.87; Figure 1).

**Figure 2:**
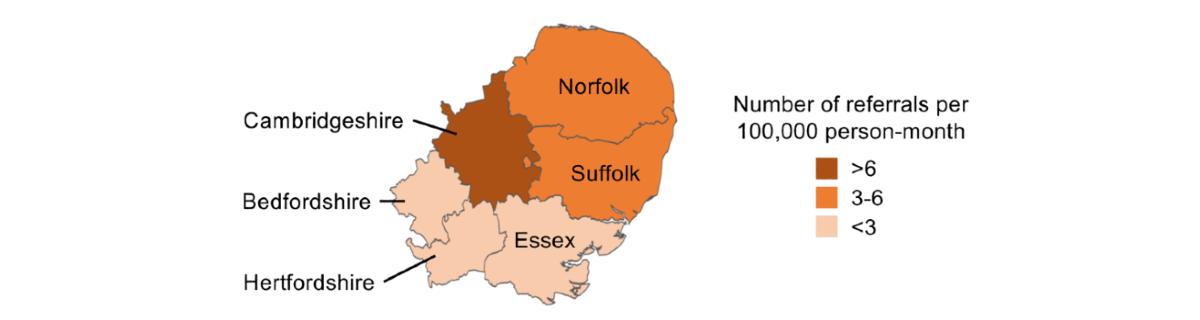
Average incidence of monthly referrals (number of referrals per 100,000 person-month) by subregion in April and September of 2016 and 2017.

Most referrals (222, 89.5%) were of patients residing within the catchment area of the study center. Within this catchment area, the average incidence of monthly referrals ranged from 0.3 (from North Essex) to 0.6 (from Cambridgeshire) referrals per 100,000 person-months (Figure 2).

Date of diagnostic imaging was missing in 36 (15%) patients, from all referral types, and for 4 (2%) patients the only available imaging occurred between referral and MDT discussion. Pre-MDT diagnostic imaging types included CT scan (125, 61.0%), MRI (72, 35.1%), or both CT and MRI scans on the same day (8, 3.9%). By referral type, MRI scans (with or without CT on the same day) were more common in paper letter referrals (12, 67%) than in the remaining referral types, including eReferrals (24, 34%), paper proforma (32, 42%), and internal referrals (12, 30%; *P*=.05).

### Referral Quality

#### Referral Efficiency

**Figure 3:**
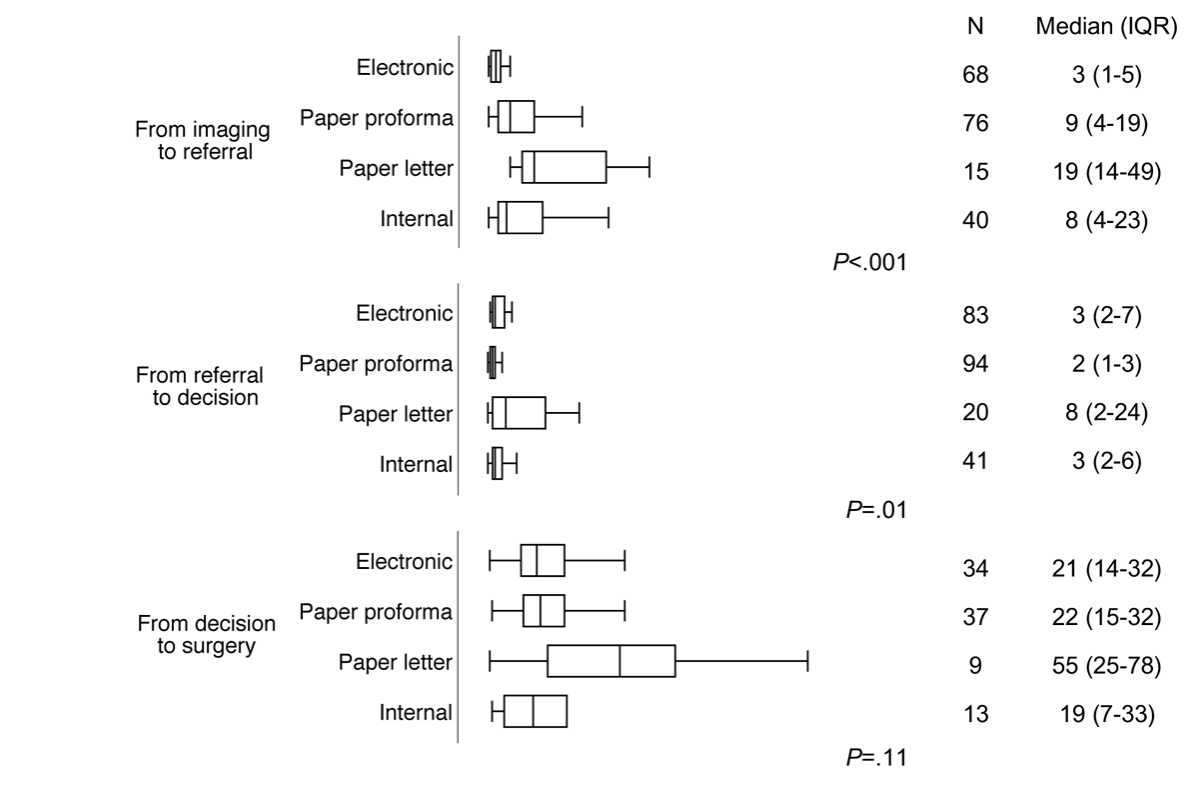
Duration of each phase of the referral pathway, during the 4 months of the study period (April and September of 2016 and 2017). Time from imaging to referral excludes 36 patients with missing date of diagnostic imaging, 4 patients with their imaging carried out after their referral, and 2 patients whose available imaging was older than 6 months before the referral. Values outside the whiskers (more than 1.5 times the IQR from the upper and lower quartiles, respectively) are not plotted. Patients with nonstandard (initially done as emergency neurosurgery referrals) or unknown referral source are not plotted (n=10). Internal refers to intrahospital referrals within the study center using the hospital’s electronic records system.

eReferrals had the shortest diagnostic imaging to-referral intervals and were, on average, 6, 16, and 5 days quicker than paper proforma, paper letter, and internal referrals, respectively (Figure 3). There was a wide variability in these intervals in all referral types with the exception of eReferrals, which were more consistent (Figure 3). In 2 patients (1 with eReferral and 1 with paper proforma referral) the interval was over 6 months, so they were considered as cases whose first diagnostic scan was missing. In all, 11 patients, from all referral types, had unexpectedly large imaging to referral intervals of 2 to 6 months. MDT diagnoses for these were mostly meningiomas (n=6, 54%) or lesion or mass (n=2, 18%), and there was 1 patient with HGG. The latter was a patient referred with a paper letter whose earliest imaging was followed by a second scan about 2 months later.

Referral to decision intervals were similar among eReferrals, paper proforma, and internal referrals, with median intervals of 2 to 3 days (Figure 3), whereas paper letter referrals were 5 to 6 days slower on average. Furthermore, 7 patients had unexpectedly large referral-to-decision intervals of over 1 month. Most of these had been referred with a paper letter (4/7, 57%) but also with paper proforma or eReferral. Diagnoses for these patients were mainly lesion or mass (3/7, 43%) or unknown, unclear, or missing (2/7, 29%), none were HGG or metastasis and most were cases that required further information or investigations followed by a rediscussion (5/7, 71%).

Among the 97 patients who would eventually have surgery, decision to surgery intervals did not differ significantly by referral type (Figure 3). In all, 5 patients, from all referral types, had unexpectedly large decision to surgery intervals of more than 3 months. Most of these had a diagnosis of meningioma (3, 60%), and none of them had HGG or metastasis.

**Figure 4:**
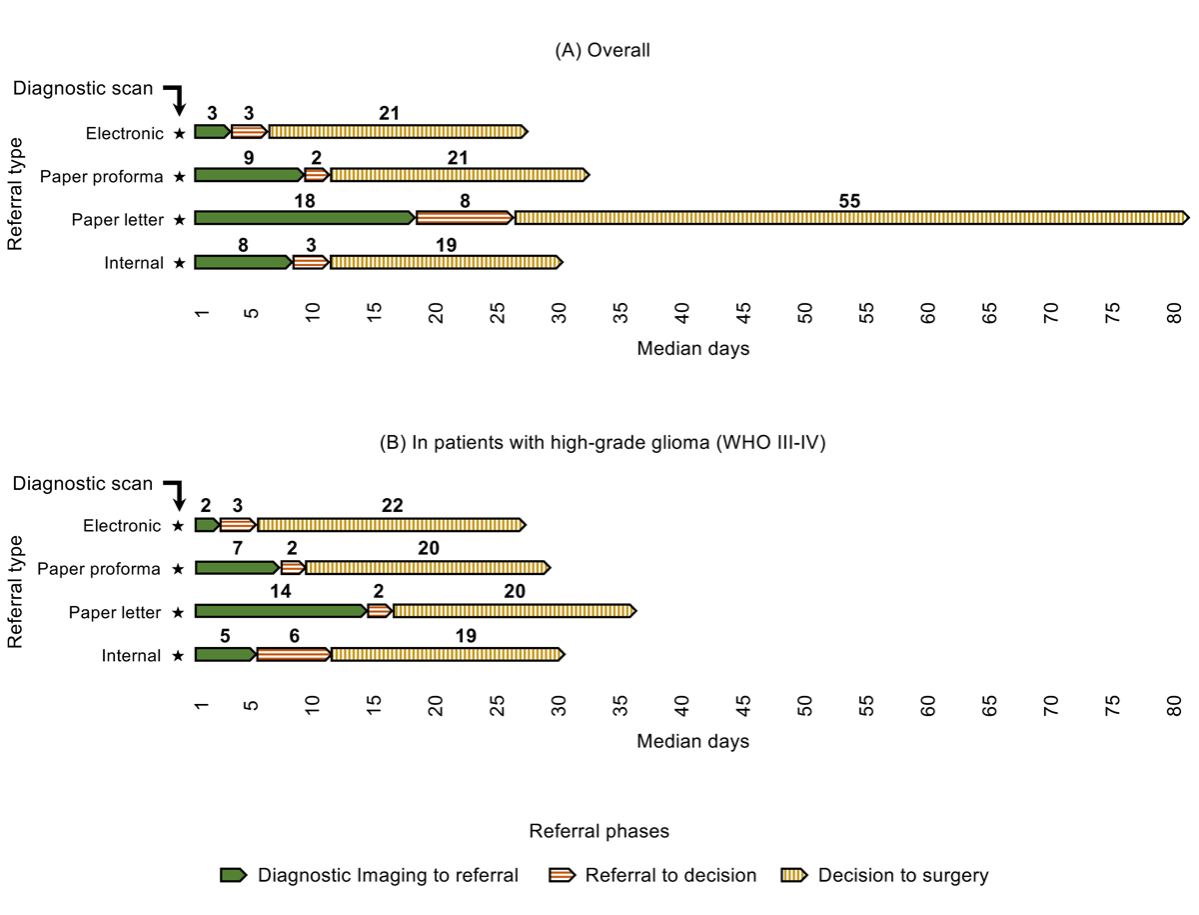
Median interval times of each referral phase, by referral type, during the 4 months of the study period (April and September of 2016 and 2017). Patients with nonstandard (initially done as emergency neurosurgery referrals) or unknown referral source are not plotted (n=10). (A) Including all patients; (B) including only patients with high-grade glioma, World Health Organization grading III-IV. WHO: World Health Organization.

A graphical representation of the sum of the median intervals at each referral pathway phase is provided in Figure 4.

Including all phases, diagnostic imaging to surgery intervals (where applicable) had median (IQR) values of 28 (21-41), 34 (27-51), 104 (69-143), and 32 (15-89) days among eReferrals, paper proforma, paper letter, and internal referrals, respectively (*P*<.001). In patients with HGGs, those values were 30 (21-41), 28 (21-33), and 37 (33-40) days in eReferrals, paper proforma, and paper letter referrals, respectively, and 85 days in the only internal referral of HGG (*P*=.32). There was no unexpectedly large diagnostic imaging to surgery interval of more than 6 months.

Subanalyses by diagnostic group (when methodologically plausible) showed that differences in diagnostic imaging to referral time remained statistically significant in all groups except meningiomas. Referral to decision time differences remained statistically significant among patients with HGGs. Subanalyses by group of month and year of MDT discussion, in each of the referral types, showed that there was no time trend in any of the time intervals of interest, which did not differ significantly by month-year group.

#### Referral Completeness

The proportion of complete fields differed significantly by referral type in all fields of interest (all with *P*<.001) except for details of presentation, which were included in all referrals, regardless of the referral source (Table 3). As expected, eReferrals had all the study fields of interest completed for 100% (n=83) of the referrals, as these were compulsory for submission. In paper proforma referrals, the percentages of complete fields ranged within 69% (65/94) to 87% (82/94), depending on the field. In the remaining referral types, these percentages showed a broader variation, ranging from between 15% (3/20) and 22% (9/41) in the staging CT field to between 63% (26/41) and 65% (13/20) in the symptom duration field (Table 3).

When looking at each diagnostic group separately, the difference in the proportion of referrals reporting each field remained statistically significant for most fields (with a few exceptions): PS in all diagnostic groups except lymphoma; symptom duration in meningiomas, metastasis, and *other CNS tumors*; previous malignancy in metastasis and *other CNS tumor*s; and in LGGs, the only field with differing reporting proportions by referral type was symptom duration.

For the 10 patients with unusual or unknown referral pathways, the median (IQR) diagnostic imaging to referral, referral to decision, and decision to surgery intervals were 4 (0-12), 2 (1-2), and 5 (3-15) days, respectively. The level of referral completeness in this group varied by the specific field considered, ranging from 20% referrals with the staging CT recorded to 100% referrals with details of presentation reported.

**Table 3:** Number and percentage of fields recorded, by referral type, excluding unusual and unknown referral pathways.

Fields recorded	Referral type^a^			
	Electronic^b^, n (%)	Paper proforma, n (%)	Paper letter, n (%)	Internal, n (%)
Performance status^c^	83 (100)	75 (80)	7 (35)	9 (22)
Details of presentation	83 (100)	94 (100)	20 (100)	41 (100)
Symptom duration^c^	83 (100)	71 (76)	13 (65)	26 (63)
Steroid treatment^c^	83 (100)	82 (87)	5 (25)	8 (20)
Previous malignancy^c^	83 (100)	74 (79)	7 (35)	23 (56)
Staging computerized tomography^c^	83 (100)	65 (69)	3 (15)	9 (22)

^a^7 referrals initially done as emergency neurosurgery referrals and three cases with unknown referral source have bene excluded.

^b^All electronic referrals were 100% complete in all fields as completing these was compulsory for the referral submission.

^c^*P*<.001.

#### Electronic Referral Software Issues

In 2017, for a total of 675 neuro-oncology surgery eReferrals carried out in the study neuroscience center, the ORION software support team received 27 calls or emails from referrers about any issues related to the use of the software, representing a maximum of 4.1% referrals requiring some type of software support. The majority of them were resolved within less than 5 min (77%) or 5 to 10 min (17%), and the nature of these calls varied, the most common ones being issues with making a new or reactivating an existing account (40%) and user errors in data entering (27%).

## Discussion

### Principal Findings

Adequate decisions and management of patients referred to specialized care depend largely on timely and high-quality written communication between referrers and specialists, often the only means of communication between both parties [4]. Our study shows that eReferrals of new patients to a specialized neuro-oncology surgery service for multidisciplinary consideration were of significantly higher quality than remaining referral types in terms of their time efficiency and completeness of information provided, which has also been suggested by studies in other clinical settings [2,12-14].

### Referral Efficiency

The time interval from diagnostic scan to a referral being received by the MDT team was 16 days shorter, on average, with eReferrals than with paper letters. This may reflect the lengthier and larger number of steps usually involved in the latter [14], whereas eReferrals in our study may just involve 2 to 4 steps, including opening ORION’s platform, creating a referrer ID (first users only), and completing and submitting the Web-based referral form. Moreover, in 2017, only about 4% of the eReferrals required some type of software support by referrers, indicating that the system was easy to use for the majority of users. In addition, the eReferral form in our study has mainly closed fields that can be answered with a single click (Multimedia Appendix 2), making its completion quicker and more straight forward than free-text letters. Furthermore, the type of diagnostic scan triggering the referral should have not affected these differences, as MRI scans, which are more specific than CT scans, were actually used more often in paper referrals than in the other types of referral. Similarly, Chen et al found that their eReferral system to different types of specialties halved the average waiting time for an initial consultative visit within 1 year, resulting in a safer and more time-efficient service [8]. A national report from Denmark found eReferrals to be faster to make and process, resulting in 15% to 30% cost savings [14].

As a form of paper referral, paper proforma referrals were also slower than eReferrals. Nonetheless, they were considerably faster than paper letter referrals, showing the usefulness of proformas to not only improve referral completeness but also to speed up the referral process.

The slower referral pathway of intra-hospital internal referrals as compared with eReferrals could be explained by cases being initially admitted under neurosurgery and subsequently referred to the MDT.

Some of the differences in the referral intervals may have been affected by more patients having more urgent diagnoses among eReferrals and internal referrals. Although not statistically significant, the proportions of patients with HGG and metastasis were higher among eReferrals and internal referrals, respectively, and the only 4 patients with WHO-PS of 4 were referred with the eReferral system.

Referral to decision intervals were also significantly longer among paper referrals, followed by eReferrals and internal referrals. This may be related to the lower completion rates in paper letter referrals, which could have made decisions more difficult.

In patients who had surgery, overall diagnostic imaging to surgery intervals were similar in all referral types, except for paper letters, where these intervals were about 3 to 4 times higher. In patients with HGGs who had surgery, paper letters were still the most inefficient, with median diagnostic imaging to surgery intervals being 7 to 9 days longer with this method. These differences identified can be a great concern in terms of the effectiveness and safety of the surgical management, especially of patients with the most aggressive and fast-growing CNS tumors. For patients with glioblastoma multiforme, the commonest HGG, tumor growth rates have been estimated at median values of 1.4% per day between the first diagnostic scan and the presurgical scan [15]. This means that, while patients with glioblastoma wait to be operated, their lesion could increase about 10% to 13% more if they are referred with traditional paper letters than if they are referred with the other referral types. Conversely, once a final MDT decision was made, the decision to surgery interval did not differ significantly by referral type, including patients with HGGs where decision to surgery intervals were 19 to 22 days in all referral types (Figure 4). This indicates that once a suspected diagnosis and potential management plan are proposed, the referral type would have not affected waiting times, and therefore, it is the intervals before referral and MDT meeting that can be affected by the referral route.

The 7 patients who had a nonstandard route of referral were usually patients who were transferred as neurosurgical emergencies before an MDT meeting. This may explain the similar diagnostic imaging to referral and referral to decision intervals, but shorter decision to surgery intervals on average.

Outlying and unexpectedly large referral intervals occurred in all referral types, which may indicate that they were not necessarily caused by the referral type only, but probably by a number of other factors. In diagnostic imaging to referral and decision to surgery intervals, these occurred mainly in patients with meningioma, in proportions that were much higher than in the overall sample, which may reflect the often-benign character and lower urgency of these cases [16,17]. In referral to decision intervals, most unexpectedly large intervals were of diagnostically inconclusive cases (ie, the diagnosis was lesion or mass or unknown or missing), which required further information or investigations followed by a rediscussion. In addition, they had been mostly referred with paper letters, which could indicate that their lower completeness found in this study made it less straight forward to make a decision.

The eReferral system also had a positive impact in making the preparation for the MDT shorter and easier. Triaging patients, or contacting referrers about outstanding investigations, became faster and simpler, as all information necessary for this was accessible electronically from the eReferral list. The real-time nature of the electronic system allowed the inclusion of patients referred up to 2 hours before the MDT, thus potentially accommodating last-minute additions that would have had to wait for a further week before discussion. Moreover, the electronic patient list generated for the MDT removed the need to prepare this manually. This means that eReferrals led to a decrease in the time and number of tasks needed for MDT preparation, which gave nurse specialists, and other professionals, more time to spend with other clinical tasks, while preserving an optimal MDT preparation process.

### Referral Completeness

It is paramount to have all necessary imaging before the MDT discussion to allow for appropriate decisions and avoid delays in treatment of brain tumors [1]. The use of structured referral sheets can improve the quality of referrals by ensuring that necessary prereferral examinations and investigations are completed before a referral [3]. Implementing such proformas electronically allowed the team to designate compulsory and fixed-option fields with appropriate validation, therefore, improving referral completeness and accuracy. In addition, the electronic format helped reducing the risks of illegibility or repeated information [2,3]. Paper proforma referrals in our study also performed well, with each field being present in 69% to 100% referrals, depending on the field, thus confirming the positive impact of using proformas, even in paper format. Conversely, key fields in unstructured paper letters were present in 35% cases or less. Such level of incompleteness in referral letters can lead to poorer decisions by referrers but also to longer referral pathways, as suggested by our findings that paper letter referrals had significantly longer referral to decision intervals, possibly because of specialists needing more time to gather all necessary information.

Similar findings have been reported in emergency neurosurgery referrals, where information in paper referrals was often missing, whereas Web-based referrals were 100% complete [2]. eReferrals have also been found to perform better in dermatology at recording identifiers and medication prescribed, although they were more incomplete than paper referrals in a number of key clinical fields [18], highlighting the value of structured eReferrals with mandatory fields.

Details of presentation was the only study prespecified key data field that was present in all referrals, regardless of the referral type, which was not surprising as this is an essential piece of information in any medical referral.

Intrahospital internal referrals also performed poorly in terms of their level of completeness. These referrals do not have a customized proforma, and, as patients are already in the hospitals’ EHR, referrers may make shorter or more incomplete referrals, maybe assuming that all the necessary information is already in the system and can be found if needed. This reinforces the need for customizing form fields to the specific referral process.

We also found that almost 90% of referrals were directly followed by a final management decision at the MDT, without the need for rediscussions, with this proportion being similar in all referral types. This figure is higher than those reported in a large US study, where the percentage of referrals immediately scheduled without any back and forth between specialist and requesting providers was 58.4% for oncology referrals and 57.7% for neurosurgery referrals [19]. This could be related to the involvement in the study center of an MDT of professionals with diverse clinical backgrounds and the virtual participation of specialists from referring hospitals, which can avoid a great proportion of rediscussions at a different MDT meeting.

### Uptake of Electronic Referrals

Since their introduction, the use of eReferrals to the study center rose steadily, and in the last month of the study period, eReferrals represented more than half of all referrals. These proportions of referrals done electronically are similar or higher than those found in similar projects in other health care settings [12,14,20], indicating the welcoming of our project by community hospitals and GPs. In addition, the distribution of referrer types remained very similar over time, indicating a low risk of provider characteristics having affected the eReferral uptake. A positive attitude of GPs toward eReferrals and health information technology systems have been shown in previous large surveys and interviews [21-23]. This has been found to be driven by referrers’ realization that eReferral systems can improve access to specialty care, and there is better appointment tracking and improved communication between referrer and specialty care providers [23,24]. In addition, eReferrals in our project keep referrers informed about the outcome of the referral and MDT discussion, the lack of which having been identified as a limitation of other eReferral systems [23].

Conversely, paper referrals were still used in our study, even a year after introduction of the eReferral system. This could be related to the fact that brain tumor patients are often diagnosed as emergencies by nonspecialist clinicians who may not be familiar with the electronic referral process. Furthermore, paper proformas remain the method of referral to the neuro-oncology MDT for the majority of UK neuroscience centers, which most referrers will be familiar with. Nonetheless, this study was carried out over the course of 18 months and the possibility that the system may not have yet achieved a *steady state* where it is operating at full potential cannot be discarded. There is evidence that resistance may arise among referrers on the use of eReferrals [13], often related to lack of developed skills or motivation around the use of technology, and unawareness of the benefits of the technology [25]. It is important that the project design involves both referrers and accepting specialty centers and it reflects the local context and addresses local barriers [3,25,26]. The electronic system for eReferrals must be disease specific and purposively developed and tailored to the needs and context of the health care setting [27]. In our study, the eReferral system implemented was designed and implemented following evidence- and experience-based recommendations for the effectiveness improvement of electronic referral communications [28]. This includes the system-collaborative development by and for the professionals who benefit directly from the system, the use of a proforma with both structured and free-text fields, the inclusion of compulsory fields such as the referral reason, and the system capabilities of being used as electronic consultation and of providing referral status tracking and feedback to referrers [27,28]. In addition, regular feedback is gathered from users by the developers, to adapt and improve the software solution based on this, which is key for a successful integration of the system too [3,26,28].

The regional variation identified in our study within the East of England was characterized by a lower monthly incidence of referrals coming from the three most southern regions. These regions are closer to London, which may have led to some cases being referred to other specialist neuroscience centers in London.

### Methodological Considerations

A number of meningiomas in our study center are not discussed as new cases but only considered as follow-ups later on, and therefore, these are underrepresented in our sample. Consequently, the distribution of diagnostic groups in our study do not reflect their reported epidemiology at the population level in similar countries [29-31].

The lower subsample size of paper letter referrals could have affected the magnitude of the differences identified. Given this, and the great variability in the study intervals identified, a future study involving a larger sample, with at least 5 patients per diagnostic group per referral type is warranted if the aim is to examine differences by tumor type.

### Conclusions

Referrals to a specialist neuroscience center, using a service-developed specific proforma, perform significantly better, in terms of time from diagnosis to referral and specialist decision, and completeness of the information provided, than free-text letters. Electronic proformas perform even better than paper proformas, through an easily accessible and structured Web form including mandatory and fixed-option fields. Complete referrals ensure that specialists receive essential information for them to be able to make optimal informed decisions about a referral and are associated with faster decisions after referrals. Faster referrals mean that time to treatment is notably shorter, thus reducing the risk of disease progression. A wider application of eReferrals within cancer services and beyond is an important first step to streamlining specialist care pathways and providing excellent care.

## Appendix

Multimedia Appendix 1 Screenshots of the eReferral portal.

Multimedia Appendix 2 eReferral form data dictionary.

Multimedia Appendix 3 Study data extraction methods and sources.

## Abbreviations

CNS: central nervous system
CT: computerized tomography
DAMSEL: Detection and Assessment of Malignancy by Symptom Evaluation
EHR: electronic hospital record
GP: general practice
HGG: high-grade gliomas
LGG: low-grade gliomas
MDT: multidisciplinary team
MRI: magnetic resonance imaging
NIHR: National Institute for Health Research
NHS: National Health Service
ORION: Outcome Registry Intervention and Operation Network
PACS: Picture Archiving and Communication System
PS: performance status
QI: quality improvement
WHO: World Health Organization
