# Supplementary material for: Improvement of the Efficiency and Completeness of Neuro-Oncology Patient Referrals to a Tertiary Center Through the Implementation of an Electronic Referral System: Retrospective Cohort Study
Source: J Med Internet Res. 2020 Mar 5;22(3):e15002. doi: 10.2196/15002 (PMC7082731; doi:10.2196/15002)
Supplement: Multimedia Appendix 1 [file jmir_v22i3e15002_app1.pdf]

Multimedia Appendix 1 – Screenshots of the eReferral portal

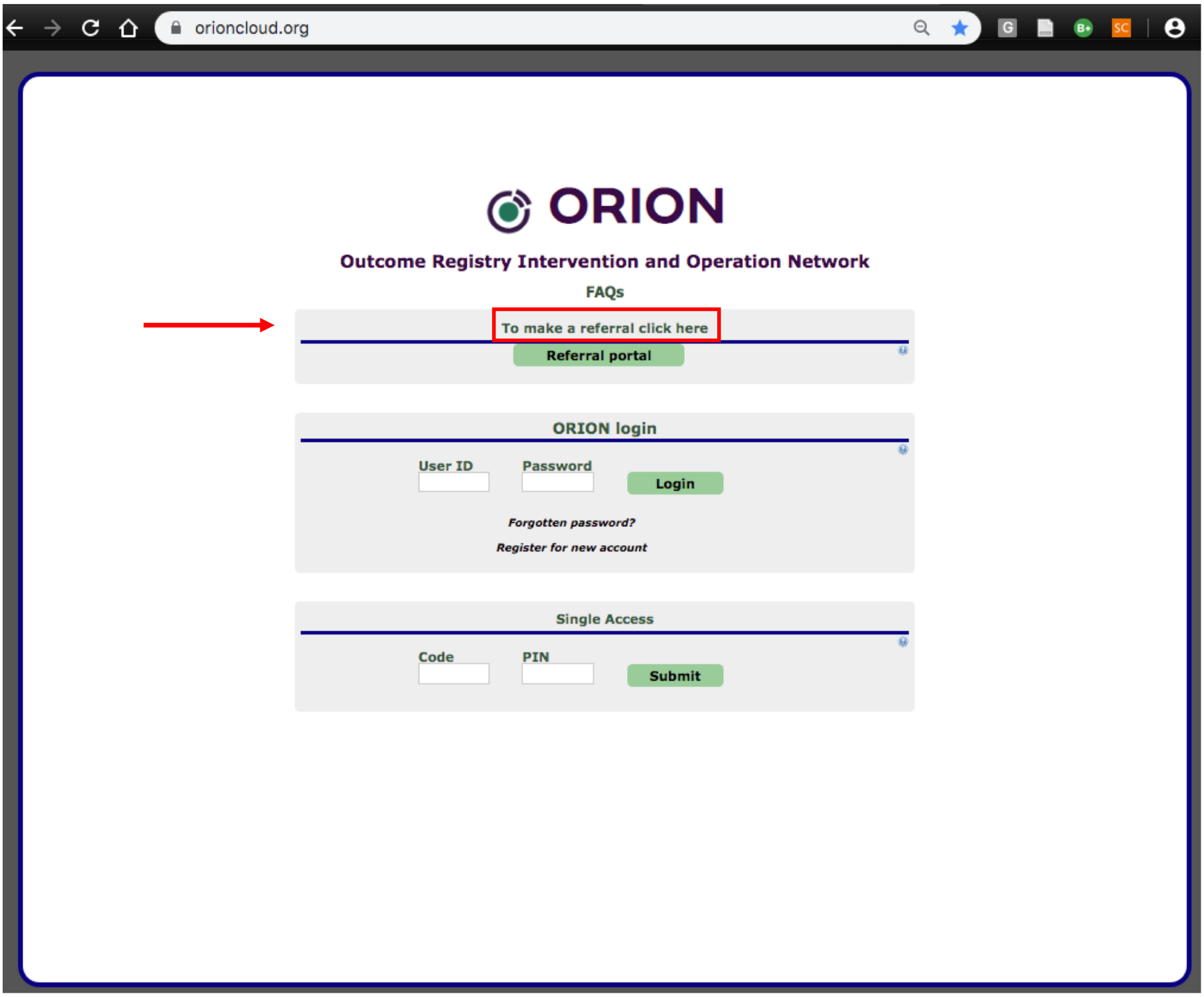

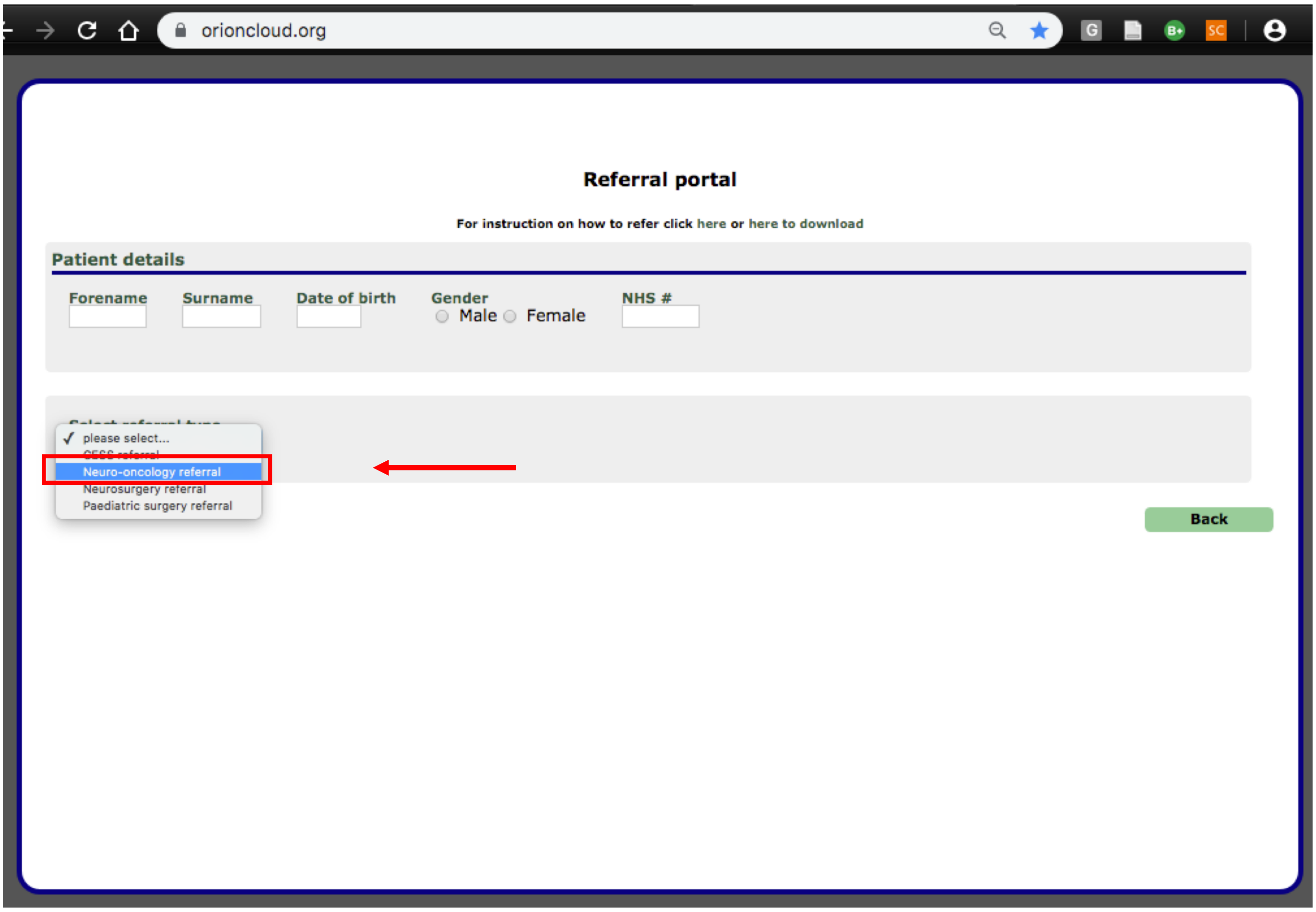

orioncloud.org

Referral portal

For instruction on how to refer click here or here to download

Patient details

Forename | Surname | Date of birth | Gender ○ Male ○ Female | NHS #

Referrer details

User ID | Password

Create new ID? | *Forgotten password?*

Neuro-oncology MDT referral form

Referral information

Referral to: please select...

Referral from ...

Designated contact

☐ I am the designated contact

Name | Email | Telephone

Responsible consultant

☐ I am the responsible consultant

Name | Email | Telephone

Clinical details

Referral type
○ New diagnosis for treatment decision ○ Return patient with progressive disease

Physical status: please select...

Smoking history: ○ Yes ○ No

Handedness: ○ Left ○ Right

Significant co-morbidities: ☐ Diabetes ☐ COPD ☐ Ischaemic heart disease ☐ Epilepsy

History
Please include details of presenting symptoms and duration, past medical history and concurrent illness as appropriate

Previous malignancy
○ Yes ○ No

Presentation

Incidental finding ☐

Presenting symptoms

☐ Cognitive impairment ☐ Seizures ☐ Dysphasia ☐ Headache

☐ Facial droop ☐ Hemiparesis ☐ Gait disturbance ☐ Visual disturbance

☐ Other

Symptoms duration: Weeks

Dexamethasone started: ○ Yes ○ No

Examination

Current GCS
E: ◯ 1 ◯ 2 ◯ 3 ◯ 4 **V:** ◯ 1 ◯ 2 ◯ 3 ◯ 4 ◯ 5 **M:** ◯ 1 ◯ 2 ◯ 3 ◯ 4 ◯ 5 ◯ 6 **GCS 15**

Confusion ◯ Yes ◯ No

Field defect ...

Dysphasia ◯ Yes ◯ No

Facial weakness ◯ Left ◯ Right ◯ No

Limb weakness ◯ Yes ◯ No

Investigations

Please ensure that, where applicable, all imaging is linked and accessible for review

Imaging performed ◯ CT ◯ MRI ◯ Both ◯ N/A

Mass lesion present ◯ Solitary ◯ Multiple ◯ Diffuse ◯ No

Staging CT performed ◯ Yes ◯ No

Performance status

◯ 0 - Fully active, able to carry on all pre-disease performance without restriction

◯ 1 - Restricted in physically strenuous activity but ambulatory and able to carry out work of a light or sedentary nature e.g., light house work, office work

◯ 2 - Ambulatory and capable of all selfcare but unable to carry out any work activities. Up and about more than 50% of waking hours

◯ 3 - Capable of only limited selfcare, confined to bed or chair more than 50% of waking hours

◯ 4 - Completely disabled. Cannot carry on any self-care. Totally confined to bed or chair

Submit

Back
