# Supplementary material for: Improvement of the Efficiency and Completeness of Neuro-Oncology Patient Referrals to a Tertiary Center Through the Implementation of an Electronic Referral System: Retrospective Cohort Study
Source: J Med Internet Res. 2020 Mar 5;22(3):e15002. doi: 10.2196/15002 (PMC7082731; doi:10.2196/15002)
Supplement: Multimedia Appendix 2 [file jmir_v22i3e15002_app2.pdf]

Multimedia Appendix 2 - eReferral form data dictionary

	ID	Field	Type	Constraints	Validation	Comments
Referral information	A1	Referral from	Fixed option	-	Mandatory	
	A2	Referral to	Fixed option	-	Mandatory	
	A3	Responsible Consultant Name	Textbox	50 characters	Mandatory	
	A4	Responsible Consultant Email	Textbox	50 characters	Mandatory	
	A5	Responsible Consultant Telephone	Textbox	50 characters	Mandatory	
	A6	I am referring on behalf of someone else	Fixed option	-		
	A7	Designated contact Name	Textbox	20 characters	Mandatory if A6 selected	Only appears if A6 selected
	A8	Designated contact Email	Textbox	20 characters	Mandatory if A6 selected	Only appears if A6 selected
	A9	Designated contact Telephone	Textbox	20 characters	Mandatory if A6 selected	Only appears if A6 selected
Clinical details	B1	Referral type	Fixed option	-	Mandatory	
	B2	Physical status	Fixed option	-	Mandatory	
	B3	Smoking history	Y/N	-	Mandatory	
	B4	Handedness	Fixed option	-	Mandatory	
	B5	Significant co-morbidities	Multiple options	-		
	B6	History	Textarea		Mandatory	
	B7	Previous malignancy	Y/N	-	Mandatory	
	B8	Primary site	Fixed option	-	Mandatory if B7=Yes	Only appears if B7 is selected
	B9	Previous treatment received	Textarea		Mandatory if B7=Yes	Only appears if B7 is selected
Presentation	C1	Incidental finding	Fixed option	-		
	C2	Presenting symptoms	Multiple options	-		Only appears if C1 is not selected
	C3	Symptom duration	Textbox	2 characters	Mandatory if C1 is not selected	Only appears if C1 is not selected
	C4	Symptom duration unit	Fixed option	-	Mandatory	Only appears if C1 is not selected, default is weeks
	C5	Dexamethasone started	Y/N	-	Mandatory	
	C6	Dexamethasone response (if >48 hours)	Y/N/NA	-		Only appears if C5=Y
Examination	D1	GCSE	Fixed option	-	Mandatory	
	D2	GCSV	Fixed option	-	Mandatory	
	D3	GCSM	Fixed option	-	Mandatory	
	D4	Confusion	Y/N	-	Mandatory	
	D5	Field defect	Fixed option	-	Mandatory	

	ID	Field	Type	Constraints	Validation	Comments
	D6	Dysphasia	Y/N	-	Mandatory	
	D7	Facial weakness	Fixed option	-	Mandatory	
	D8	Limb weakness	Y/N	-	Mandatory	
	D9	Left upper limb power	Fixed option	-	Mandatory if D8=Y	Only appears if D8=Y
	D10	Right upper limb power	Fixed option	-	Mandatory if D8=Y	Only appears if D8=Y
	D11	Left lower limb power	Fixed option	-	Mandatory if D8=Y	Only appears if D8=Y
	D12	Right lower limb power	Fixed option	-	Mandatory if D8=Y	Only appears if D8=Y
Investigations	E1	Imaging performed	Fixed option	-	Mandatory	
	E2	Mass lesion present	Fixed option	-	Mandatory	
	E3	Contrast enhancing	Y/N/NA	-	Mandatory if E2=1,2 or 3	Only appears if E2=1,2 or 3
	E4	Location	Multiple options	-		Only appears if E2=1,2 or 3
	E5	Imaging findings	Textarea		Mandatory if E1=1,2 or 3	
	E6	Staging CT performed	Y/N	-	Mandatory	
	E7	Abnormality identified	Y/N	-	Mandatory if E6=Y	Only appears if E6=Y
	E8	Staging CT findings	Textarea		Mandatory if E7=Y	Only appears if E7=Y
Performance status	F1	Performance status	Fixed option	-	Mandatory	
Management	G1	Plan	Textarea	-	Mandatory	Only appears for private referrals
	G2	Referral outcome	Fixed option	-	Mandatory	Only appears for private referrals
