# Supplementary material for: Improvement of the Efficiency and Completeness of Neuro-Oncology Patient Referrals to a Tertiary Center Through the Implementation of an Electronic Referral System: Retrospective Cohort Study
Source: J Med Internet Res. 2020 Mar 5;22(3):e15002. doi: 10.2196/15002 (PMC7082731; doi:10.2196/15002)
Supplement: Multimedia Appendix 3 [file jmir_v22i3e15002_app3.pdf]

Multimedia Appendix 3 – Data extraction methods and sources

Variable label	Electronic referrals: Extraction method	Electronic referrals: Source	Other referrals: Extraction method	Other referrals: Source
Sex	Automatic	Orion	Manual	EHR
Age on referral	Automatic	Orion	Manual	EHR
Postcode	Automatic/ Manual	Orion/EHR	Manual	EHR
Performance status on referral	Automatic	Orion	Manual	EHR
Referring hospital	Automatic	Orion	Manual	EHR
Referral reason	Automatic	Orion	Manual	EHR
Diagnosis after MDT discussion	Manual	EHR	Manual	EHR
Eventual diagnosis	Manual	EHR	Manual	EHR
Date of referral pre-MDT	Automatic	Orion	Manual	EHR
Date of emergency neurosurgical referral pre-MDT	Automatic	Orion	Manual	EHR
Date of pre-MDT imaging CT	Manual	PACS	Manual	PACS
Date of pre-MDT imaging MRI	Manual	PACS	Manual	PACS
Date of MDT discussion	Manual	EHR	Manual	EHR
Date of decision	Manual	EHR	Manual	EHR
Date of re-discussion	Manual	EHR	Manual	EHR
Date of neurosurgery (if any)	Manual	EHR	Manual	EHR
Address recorded (y/n)	Automatic	Orion	Manual	EHR
Performance status stated recorded (y/n)	Automatic	Orion	Manual	EHR
Details of presentation recorded (y/n)	Automatic	Orion	Manual	EHR
Symptom duration recorded (y/n)	Automatic	Orion	Manual	EHR
Steroid treatment recorded (y/n)	Automatic	Orion	Manual	EHR
Previous malignancy recorded (y/n)	Automatic	Orion	Manual	EHR
Staging CT recorded (y/n)	Automatic	Orion	Manual	EHR
Outcome from MDT discussion	Manual	EHR	Manual	EHR
Reason for re-discussion	Manual	EHR	Manual	EHR
Reason for no decision	Manual	EHR	Manual	EHR
Comments to any of the variables	Manual	EHR	Manual	EHR

EHR: Electronic Hospital Records system; Orion: Outcome Registry Intervention and Operation Network platform for electronic referrals; PACS: Picture Archive Communication system of the Electronic Hospital Records system
